# 4328 Translational Fellows as a mechanism to improve throughput of university technology commercialization

**DOI:** 10.1017/cts.2020.162

**Published:** 2020-07-29

**Authors:** Everett Gordon Hall, Tom Krenning, Michael Seper, Michael Kinch

**Affiliations:** 1 Washington University in Saint Louis; 2 Skandalaris Center, Washington University in Saint Louis

## Abstract

OBJECTIVES/GOALS: The development of early university technologies for commercialization is largely inefficient and exhibits a high rate of failure, often due to a lack of researcher time and commercialization experience. We have created the Translational Fellow role to address these needs and increase the throughput of university technology transfer. METHODS/STUDY POPULATION: Translational Fellows will first build their initial competencies to identify, evaluate, and develop new technologies through internships with intake organizations within the university ecosystem, including the Office of Technology Management, the LEAP gap-funding mechanism, and local venture capital firms. Following this training, Fellows will provide tailored support to validated projects by establishing development milestones, liaising with industry experts, navigating regulatory requirements, and drafting marketing materials such as executive summaries and financial projections. Lastly, Fellows will partner with a highly developed project to facilitate the commercialization of the technology, whether through a SBIR/STTR grant, direct licensing event, or startup creation. RESULTS/ANTICIPATED RESULTS: We anticipate that implementation of this mechanism will increase the proportion of university-generated inventions that undergo successful commercialization events, as well as increase the rate at which these projects develop after initial validation. Furthermore, we expect that the skills acquired through this program will allow Fellows to successfully transition to a variety of roles in the biotech space. We also expect that Fellows will be capable of training other scientific teams in the preparation of SBIR/STTR grants, further expanding opportunities for commercialization in the research space. DISCUSSION/SIGNIFICANCE OF IMPACT: Translational Fellows fill a unique interdisciplinary niche, allowing them to address common barriers faced by academic inventors. Improving commercialization throughput further capitalizes on the wealth of ideas generated in universities, thereby driving innovation in the biomedical space and directly contributing to improved human health. CONFLICT OF INTEREST DESCRIPTION: The authors have no conflicts of interest.

